# Methods to identify the prey of invertebrate predators in terrestrial field studies

**DOI:** 10.1002/ece3.2791

**Published:** 2017-02-22

**Authors:** Klaus Birkhofer, Helena Bylund, Peter Dalin, Olga Ferlian, Vesna Gagic, Peter A. Hambäck, Maartje Klapwijk, Laia Mestre, Eve Roubinet, Martin Schroeder, Johan A. Stenberg, Mario Porcel, Christer Björkman, Mattias Jonsson

**Affiliations:** ^1^ Department of Biology, Biodiversity and Conservation ScienceLund UniversityLundSweden; ^2^ Chair of EcologyBrandenburg University of TechnologyCottbus‐SenftenbergGermany; ^3^ Department of EcologySwedish University of Agricultural SciencesUppsalaSweden; ^4^ German Centre for Integrative Biodiversity Research (iDiv) Halle‐Jena‐LeipzigLeipzigGermany; ^5^ Institute of BiologyLeipzig UniversityLeipzigGermany; ^6^ CSIROBrisbaneQLDAustralia; ^7^ Department of Ecology, Environment and Plant SciencesStockholm UniversityStockholmSweden; ^8^ Ecosystem AnalysisInstitute for Environmental SciencesUniversity of Koblenz‐LandauLandauGermany; ^9^ Department of Plant Protection BiologySwedish University of Agricultural SciencesAlnarpSweden

**Keywords:** cage experiments, fatty acid analysis, field observations, molecular gut content analysis, prey baits, stable isotope analysis

## Abstract

Predation is an interaction during which an organism kills and feeds on another organism. Past and current interest in studying predation in terrestrial habitats has yielded a number of methods to assess invertebrate predation events in terrestrial ecosystems. We provide a decision tree to select appropriate methods for individual studies. For each method, we then present a short introduction, key examples for applications, advantages and disadvantages, and an outlook to future refinements. Video and, to a lesser extent, live observations are recommended in studies that address behavioral aspects of predator–prey interactions or focus on per capita predation rates. Cage studies are only appropriate for small predator species, but often suffer from a bias via cage effects. The use of prey baits or analyses of prey remains are cheaper than other methods and have the potential to provide per capita predation estimates. These advantages often come at the cost of low taxonomic specificity. Molecular methods provide reliable estimates at a fine level of taxonomic resolution and are free of observer bias for predator species of any size. However, the current PCR‐based methods lack the ability to estimate predation rates for individual predators and are more expensive than other methods. Molecular and stable isotope analyses are best suited to address systems that include a range of predator and prey species. Our review of methods strongly suggests that while in many cases individual methods are sufficient to study specific questions, combinations of methods hold a high potential to provide more holistic insights into predation events. This review presents an overview of methods to researchers that are new to the field or to particular aspects of predation ecology and provides recommendations toward the subset of suitable methods to identify the prey of invertebrate predators in terrestrial field research.

## 1 Introduction

Predation is a biological interaction during which one organism kills and feeds on another organism and therefore shapes natural and anthropogenic ecosystems. For example, the loss of apex predators from terrestrial ecosystems causes significant changes in vegetation composition and structure due to herbivore prey being released from predation (Estes et al., 2011). Fundamental concepts in ecology are therefore centered on trophic interactions between predators and prey, for example keystone predation (Harley, 2011) or trophic cascades (Schmitz, Hamback, & Beckerman, 2000). Predators provide crucial ecosystem services to human societies, as they reduce or control the damage caused by herbivores in natural and managed habitats (Costanza et al., 1997), suppress vectors of human diseases (Raghavendra, Barik, Reddy, Niranjan, & Dash, 2011), and conserve natural ecosystems (Sergio et al., 2008). Past and current interests in predation events have yielded an impressive number of methods to assess predation in field studies of terrestrial habitats. These approaches differ in their ability to quantify predation, to identify behavioral aspects of predator–prey interactions and in their suitability for specific systems or research questions. Due to such limitations, specific methods are more or less suitable to address different research topics such as qualitative or quantitative food webs, biological control of pests or prey selection behavior. Existing reviews cover fairly novel approaches (DNA‐based, stable isotope, or fatty acid analyses, Traugott, Kamenova, Ruess, Seeber, & Plantegenest, 2013), review subsets of methods such as cage experiments (Schmitz, 2004) and video observations (Chisholm, Gardiner, Moon, & Crowder, 2014), or focus on the impact of natural enemies on pest prey (Macfadyen, Davies, & Zalucki, 2015).

**Figure 1:**
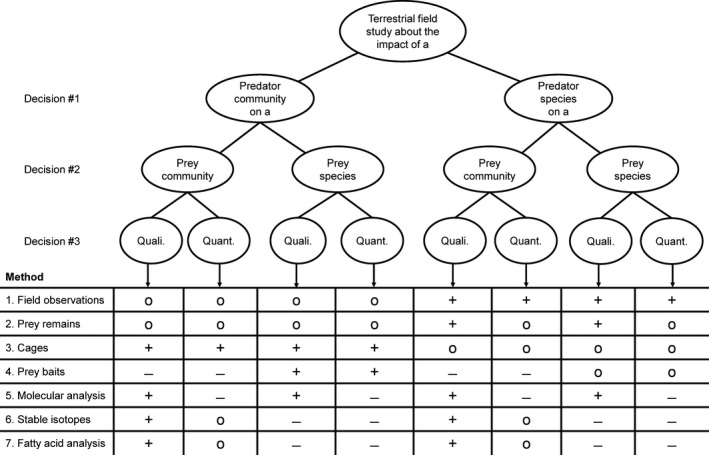
Decision tree leading to the major domains of methods to measure predation in terrestrial field studies. Decision #1 addresses whether the focus is a single or very few predator species or a whole community of predators. Decision #2 addresses whether the whole prey community is of interest or whether predation on a single species is assessed. Decision #3 addresses the need for qualitative (link between prey present or absent) or quantitative data on predation. The table then illustrates the suitability of each method for that particular domain with +, suitable in most cases; ○, suitable in some cases and – unsuitable in most cases. After a subset of methods is selected from this figure, please refer to Table 1 for additional suitability criteria of individual methods for different body size and mobility traits of predators

Here we provide a comprehensive overview of methods to qualitatively and quantitatively assess invertebrate predation in field studies of terrestrial ecosystems with a focus on identifying predator–prey interactions and their impact on prey populations. This overview updates the existing sources that previously addressed the majority of methods for invertebrate predators (Jervis, 2005; Luck, Shepard, & Kenmore, 1988) and extends the scope to include a wider range of methods. Figure 1 shows the suitability of methods in relation to the level of predator and prey identity needed in a study (community vs. single species level), the methods’ ability to provide qualitative or quantitative predation data, and predator traits such as body size and mobility. After identifying a suitable subset of methods by using Figure 1, the text presents a short introduction, examples for appropriate study questions, advantages and disadvantages, and an outlook to future refinements for each major method. Key references are provided to direct researchers to important papers that document details on the application of methods and the analysis of data. We therefore aim to provide an overview for researchers and to facilitate selection of methods for future field studies that aim at identifying the prey of invertebrate predators in terrestrial ecosystems.

## 2 Field Observations

Field observations of predation events are the most direct approach to assess a predator species’ diet breadth, prey diversity, and predation pressure on selected prey species (Sih, Crowley, McPeek, Petranka, & Strohmeier, 1985). A full assessment of predation events between communities of invertebrate predators and their prey is often compromised by the cryptic nature of predation events between certain predator–prey combinations in local communities (Figure 1). Because these data are collected during the predation event, they additionally provide information on the predators’ behavior and feeding habits (Björkman, Dalin, & Eklund, 2003) and temporal predation patterns. In addition to direct field observations, video surveillance of predator–prey interactions has been common, particularly in studies of larger predator species (Varley, Copland, Wratten, & Bowie, 1994).

### 2.1 Applications and advantages and disadvantages

#### 2.1.1 Live observations

**Figure 2:**
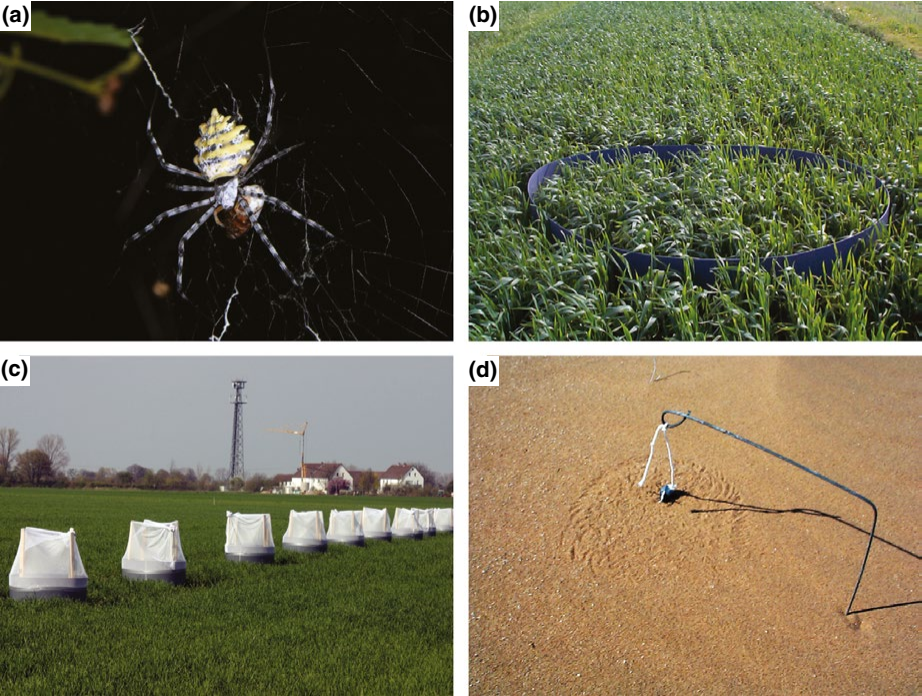
Examples of different methods to assess predation events in the field with (a) field observations, (b) exclosure barrier, (c) enclosure cage, and (d) sentinel prey (darkling beetle on a string)

Sunderland (1988) provides a comprehensive overview of field studies focusing on the observation of predation events between invertebrates (e.g., spiders, Figure 2a), highlighting some of the major characteristics that make a study system suitable for this approach: (1) when prey are exposed and have limited mobility, (2) where observations are possible without disturbing prey or predator behavior, (3) when prey and predator can be identified to a required taxonomic level by observation. Data from live observations of predation events and estimates of predator abundances can be combined to calculate daily, area‐based predation rates (Jervis, 2005). Greenstone (1999) listed field‐recorded rates from previously published studies on spider–prey interactions and values ranged from 5.8 to 0.01 observed predation events per person hour. Live observations can introduce an observer bias leading to uncharacteristic predator or prey behavior, and particularly for smaller and more mobile predator or prey species predation rates will be underestimated.

#### 2.1.2 Video surveillance

Video surveillance of invertebrate predation has been used to study the effect of prey density on predation (Schenk & Bacher, 2002) and to compare predation on prey between vegetation strata (Frank, Wratten, Sandhu, & Shrewsbury, 2007). This method is especially useful for the identification of dominant predators (Schenk & Bacher, 2002), detection of unexpected predation events (Grieshop et al., 2012), or to explore systems for which little information about predator–prey interactions is available (Chisholm et al., 2014). Individual predator behavior is recorded for each encounter, and this allows establishing variables such as attack rates, predation efficiency, or handling time (Frank et al., 2007; Schenk & Bacher, 2002). The predation event must be located within the camera range, and this limits the methodology to systems involving sessile or sentinel prey or predators (Table 1). An additional constraint under field conditions is the establishment of video recording equipment which may require permits and suffers from a risk of theft.

**Table 1:** Overview of individual methods to study predation in terrestrial invertebrate and vertebrate predators in the field

Method	Predators					Estimates
	Small (many Invertebrates)	Medium (many birds or reptiles)	Large (many mammals)	Mobile predators	Sedentary predators	
1. Field observations						
Live observations	○	+	+	○	+	Prey items per predator, prey spectrum, attack rate, success rate
Video surveillance	○	+	○	○^a^	+	
2. Prey remains						
Gut content	○	+	+	+	+	Prey spectrum
Collected prey	○	+	+	−	+	Prey items per predator, prey spectrum
3. Cages						
Exclosures	+	+	○	+	+	Predator impact on prey population
Enclosures	+	−	−	−	+	
4. Prey baits						
Artificial prey	○	+	−	+	−	Attack rates
Sentinel prey	+	+	−	+	−	Predator impact on prey population
5. Molecular analysis						
Diagnostic PCR	+	+	+	+	+	Commonness of prey in predator diet, prey spectrum
DNA sequencing	+	+	+	+	+	
6. Stable isotopes	+	+	+	○^a^	+	Major prey source
7. Fatty acid analysis	+	+	+	+	+	Major prey source

The predator size range and mobility categories indicate whether methods are particularly suitable for certain predator species or not. The column “Estimates” states examples of metrics that can be derived from the application of a particular method. A method is particularly suitable (+), only moderately suitable (○), or not suitable (−) for a specific predator trait.

Suitable if prey is sedentary.

a Issues with finding valid isotope baselines.

### 2.2 Future refinements and improvements

Although video surveillance is a widely used method for the assessment of predation in vertebrate systems, the potential for the study of invertebrate predation is rather underexploited (Varley et al., 1994). Technological advancements and future developments are likely to further facilitate the implementation of the methodology. Grieshop et al. (2012) suggested that video surveillance techniques would be particularly useful in combination with methods that can produce highly replicated data. Combining field observations or video surveillance footage of predation events, with data on predator–prey population dynamics and experimental evaluation of effects on prey populations, holds great potential (e.g., Rutledge, O'Neil, Fox, & Landis, 2004).

## 3 Prey Remains

Prey remains can be studied from an invertebrate predator's gut content or regurgitates (Sunderland, 1988). Invertebrate predators that use trapping devices (e.g., web‐building spiders, Nyffeler, 1999) or collect prey in a central foraging place (e.g., burrow‐living spiders, Henschel, 1994) offer another opportunity to study predator diets. These methods are most suitable to study the diet in a single or few focal predator species with an emphasis on identifying predator–prey links in general (qualitative, Figure 1).

### 3.1 Applications and advantages and disadvantages

#### 3.1.1 Gut content and feces

The analysis of prey remains in gut content or feces in invertebrate predators has been used mainly in carabid beetles (Hengeveld, 1979). The sampling of gut content is destructive (if not using regurgitates), prey remains can often only be identified to a coarse taxonomic level, and this method is restricted to predator species without extraintestinal digestion and prey species with hard body parts that are suitable for identification. Reconstructing the diet from consumed prey remains further causes biased estimates due to known problems with differential digestibility of body parts (Speakman, 1991).

#### 3.1.2 Collected remains

Predators that collect prey remains or leave skeletal remains at a feeding site offer an opportunity to study their prey spectrum. Central place foragers may offer the best system to study predation by analyzing collected prey remains. Web‐building spiders, for example, collect remains that offer information on environmental (Diehl, Mader, Wolters, & Birkhofer, 2013) and global (Birkhofer & Wolters, 2012) drivers of diet breadth. These analyses are often constrained to coarse taxonomic resolutions of prey (e.g., order level) and smaller, less sclerotized prey will be underestimated.

### 3.2 Future refinements and improvements

Methods to analyze prey remains in gut content in terrestrial ecosystems have now largely been replaced by molecular methods to detect prey DNA. The analysis of collected remains, however, still allows quantifying the number of prey items consumed, which is an advantage over molecular methods (see section 3.1).

## 4 Cage Experiments

Cage experiments include predator exclusion and enclosure treatments. Predator exclusions involve placing cages or barriers around study areas during a specific time period to estimate predation pressure under field or semifield conditions. Predator enclosures also make use of cages or barriers, but predator communities inside are experimentally manipulated. The response variable in most cage experiments is change in prey abundance under the presence or absence of certain predator groups compared to an unmanipulated treatment. The outcome of cage experiments is then a consequence of direct predator–prey interactions as well as indirect effects resulting from predator and prey manipulations. These methods are most suitable to address the joint effect of predator communities or functional groups on prey populations, as species‐level manipulations of predators are difficult, but not impossible, to establish and to maintain (Figure 1).

### 4.1 Applications and advantages and disadvantages

#### 4.1.1 Exclusion cages or barriers

Experiments combining one or more exclusion treatment with unmanipulated controls are a powerful tool to quantify the impact of predators on prey (Rusch, Bommarco, Jonsson, Smith, & Ekbom, 2013; Schmidt et al., 2003). Exclusion cages or barriers (Figure 2b) can be constructed to selectively exclude flying or ground‐dwelling predators (Schmidt et al., 2003) or predators of different body size (Romeu‐Dalmau, Espadaler, & Pinol, 2010). Exclusions are often placed around a small area of plants with either natural (Östman, Ekbom, & Bengtsson, 2003) or standardized invertebrate prey densities (Rusch et al., 2013). A combination of different exclusion treatments allows quantification of additive and interactive effects of different predator groups (Martin, Reineking, Seo, & Steffan‐Dewenter, 2013). Exclusion experiments will work best with sessile prey and exclusion cages and barriers should be regularly checked to make sure that the target taxa are completely excluded (Ameixa & Kindlmann, 2011). Being limited to the exclusion of broad functional groups of natural enemies, this method has a relatively low specificity in disentangling effects of individual predator species (but see Mestre, Piñol, Barrientos, & Espadaler, 2016) and will introduce bias by hindering the movement of predator and prey species (Schmitz, 2004).

#### 4.1.2 Enclosure cages or barriers

Enclosures aim to restrict certain predators or prey to an area inside an enclosure and can range in size from Petri dishes to field cages covering several m^2^ (Figure 2c). The main advantage of enclosures is that the composition and abundance of both predators and prey can be manipulated (Lang, Filser, & Henschel, 1999). Recent studies have focused on the impact of single predator species on a selected target prey (Bahar, Stanley, Gregg, Del Socorro, & Kristiansen, 2012) or the full range of prey taxa (Birkhofer, Fliessbach, Wise, & Scheu, 2008), and on the impact of different predator functional groups on a selected prey (Birkhofer, Gavish‐Regev et al., 2008). Enclosures affect the behavior of predators and prey by restricting movement and are therefore mainly suitable for short‐term experiments. Cages may also alter the microclimate, and it is crucial to select cage sizes that limit effects on the behavior of predators and prey (Björkman et al., 2003) and to consider control treatments for cage effects (Schmitz, 2004).

### 4.2 Future refinements and improvements

Data from cage studies can be used for the analysis of predator communities using ecological traits and these approaches provide important insights to the community properties that determine predator–prey interactions and predation rates (Gagic et al., 2015). A challenge for future cage experiments is to manipulate the composition of traits within a predator guild, for example by using a full factorial experiment that selectively excludes/includes predaceous species with different body sizes or activity periods. Enclosure experiments are particularly suitable for such manipulations as they allow for testing the effects of predators with specific traits individually and in combination with predators with alternative traits.

## 5 Prey Baits

Prey baits include sentinel prey as individuals that are glued or tethered to a substrate (Kneib & Scheele, 2000) or artificial prey items made from plasticine or clay and resembling real prey in size, color, and shape (Howe, Lovei, & Nachman, 2009). Both types of prey baits are exposed to predators under field conditions, and the frequency of prey removal and mortality (sentinel prey) or the number and type of predator marks (artificial prey) are then recorded. These methods are most suitable to assess predation of predator communities on a single, focal prey type (Figure 1).

### 5.1 Applications and advantages and disadvantages

#### 5.1.1 Artificial prey

Artificial prey has been used to estimate predation rates by arthropod predators in forest (e.g., Tvardikova & Novotny, 2012) and agricultural (Howe, Nachman, & Lovei, 2015; Howe et al., 2009) ecosystems. The relative contribution of different predator types to the rates of predation has been compared under various habitat conditions, for example in landscapes along a gradient of surrounding natural habitat (Lemessa, Hamback, & Hylander, 2015). Advantages of artificial prey are that these methods may provide estimates of attack rates and that they are relatively inexpensive. Currently, photograph identification databases are available (Howe et al., 2009); however, even with pictures, identification of predators is not entirely objective and suffers from a coarse taxonomic resolution for predators and a bias due to the lack of realistic chemical, tactile or vibratory cues from prey (Howe et al., 2009; Low, Sam, McArthur, Posa, & Hochuli, 2014). The lack of these characteristics, particularly the absence of prey movement, further complicates the interpretation of results, as it is rather scavenging if a motionless prey is attacked.

#### 5.1.2 Sentinel prey

Sentinel prey can be used to assess the numerical impact of predators on a few selected prey species. Geiger et al. (2010) used glued aphids to show that biocontrol efficacy was lower in farms exposed to insecticides as compared to less exposed farms (see also Winqvist et al., 2011). Kessler and Baldwin (2001) used glued herbivore eggs to show that tobacco plants emitting herbivore‐induced plant volatiles attract predators from a distance and obtain a higher predation pressure compared to control plants. The defensive behavior and general appearance of mobile prey, however, are altered and constrained if glued or tethered; therefore, prey may become more or less susceptible to predators (Figure 2d). Gluing or pinning a substrate to which the prey is already naturally attached, instead of attaching the prey itself, may partly overcome problems with altered behavior that have been described from tethered prey (Kneib & Scheele, 2000). Pinning can induce the production of volatile organic compounds in the experimental plant, leading to unintended attraction of predators, and a higher‐than‐natural predation rate. Gluing, on the other hand, does not necessarily affect plants, but glue may emit chemical components that affect predators or prey, although this can partly be avoided using wallpaper paste (e.g., Kessler & Baldwin, 2001).

### 5.2 Future refinements and improvements

If standardized protocols are developed, we anticipate that new applications for sentinel and artificial prey will open up in the future. Morphometric quantifications of bite marks caused by different predator species could be used to compile online reference databases for specific systems (Low et al., 2014). Recent studies further emphasize that the selection of materials for artificial prey (Sam, Remmel, & Molleman, 2015), color patterns (Karpestam, Merilaita, & Forsman, 2014), and olfactory cues (Koski et al., 2015) affect predation estimates.

## 6 Molecular Gut Content Analysis

Molecular techniques allow the detection of prey‐specific molecules in predator regurgitates, gut contents or feces (Symondson, 2002). Early works relied on the detection of protein markers using isoenzyme electrophoresis (Paill, Backeljau, Grimm, Kastberger, & Kaiser, 2002) or monoclonal antibodies in predatory arthropod guts (Ragsdale, Larson, & Newsom, 1981). Serological techniques allowed the detection of stage‐specific prey, and were for long favored to screen large numbers of predators for a single prey species (Fournier, Hagler, Daane, de Leon, & Groves, 2008). Since the late 1990s, methods based on DNA using polymerase chain reaction (PCR) have been developed for the detection of prey in predator guts (Traugott et al., 2013). In recent years, the development of DNA bar coding has offered the possibility to identify the complete predator diet by simultaneously amplifying and sequencing DNA from all organisms present in a sample (Pompanon et al., 2012). These methods are suited for studies that address predation across a range of predator or prey taxa, but currently do not provide quantitative estimates for these links (but see section Diagnostic PCR, Figure 1).

### 6.1 Applications and advantages and disadvantages

#### 6.1.1 Diagnostic PCR

Diagnostic PCR has improved from targeting one prey species (“singleplex” PCR assays) to detecting DNA of multiple prey species simultaneously (“multiplex” PCR assays) (King et al., 2011). The use of molecular techniques has contributed to the identification of key predators of pest species (Kuusk, Cassel‐Lundhagen, Kvarnheden, & Ekbom, 2008), the understanding of the use of alternative nonpest prey by predators (King et al., 2011), recordings of intraguild predation (Davey et al., 2013), and the identification of predator niches that are particularly vulnerable to environmental change (Clare, 2014). Studies have investigated how factors such as pesticide application, landscape change, agricultural management, and changes in predator community composition affect predator–prey interactions (Birkhofer, Gavish‐Regev et al., 2008; Furlong, 2015; Roubinet et al., 2015). The detection of prey DNA in diagnostic PCR relies on a priori knowledge about the presence of prey taxa in the system and yields binary data on the presence or absence of prey DNA in predators. Semiquantitative data can be deducted using the proportion of predators for which prey DNA was detected (Davey et al., 2013; Roubinet et al., 2015). Limitations of prey detectability are acknowledged in the literature (King, Read, Traugott, & Symondson, 2008; Piñol, San Andres, Clare, Mir, & Symondson, 2014): The detection of minute amounts of DNA due to contamination in PCR‐based methods can cause false positives, differentiation between predation and scavenging or secondary predation is not possible, and cannibalism cannot be assessed by DNA‐based molecular assays. The difference in time after which prey is still detectable in different predator species (detectability half‐life) is another issue that requires attention if different predator–prey combinations are compared (Greenstone, Payton, Weber, & Simmons, 2014).

#### 6.1.2 DNA bar coding and next‐generation sequencing (NGS)

DNA bar coding is a promising tool to investigate the complete diet width and composition of predators and does not rely on any a priori assumptions about feeding links (Varennes, Boyer, & Wratten, 2014). Such sequence‐based identification of prey requires reliable databases including all organisms of the studied systems, but information on genus or family level is available for most prey groups in temperate areas. A current limitation is also the need for bioinformatic skills to handle the data (Pompanon et al., 2012), but this problem will be reduced with the development of analysis pipelines. Databases can either be developed specifically for the study area, at a fairly low cost, by building a library of sequences for potential prey species or by using common bar coding sites such as CO1. In both cases, it is important to select primers that amplify prey but not the predator. Using this method, Wirta et al. (2015) quantified a large part of the food web in an Arctic ecosystem and species identification was possible through targeted bar coding of potential prey species. An alternative is to use blocking primers that exclude amplification of predator DNA (Deagle, Kirkwood, & Jarman, 2009), but this approach may have problems in identifying prey that is taxonomically related to the predator. To resolve this problem, Piñol et al. (2014) suggested using very general primers and remove predator sequences bioinformatically afterward.

### 6.2 Future refinements and improvements

The amount of DNA in a predator sample can be quantified using semiquantitative methods, such as real‐time PCR or quantitative PCR. However, this amount is a function of multiple factors such as size of DNA fragment targeted, temperature, quantity of target prey (and of nontarget prey) ingested, time from ingestion, and number of primer–template mismatches. These problems vary in importance but precise quantification of predation for individual predators still remains problematic (Piñol, Mir, Gomez‐Polo, & Agusti, 2015). The development of novel molecular tools as well as a rapid decline in costs is likely to make molecular gut content analysis a standard tool in future predation studies.

## 7 Stable Isotope Analyses

Stable isotope analyses (SIA) of ratios of nitrogen (^15^N/^14^N = δ^15^N) and carbon (^13^C/^12^C = δ^13^C) are commonly used to describe the trophic structure of communities (Boecklen, Yarnes, Cook, & James, 2011; Layman, Arrington, Montana, & Post, 2007). Trophic discrimination of δ^15^N is used to determine the trophic level of organisms relative to some baseline, because the heavy isotope accumulates up the food chain, whereas discrimination of δ^13^C is negligible and retrieves the signal of the base of the food web, allowing the characterization of dietary sources (Post, 2002). This method is particularly suitable for predation studies that address links between predators and groups of prey from different trophic levels or with different basal resources (Figure 1).

### 7.1 Applications and advantages and disadvantages

#### 7.1.1 Naturally occurring stable isotopes

Predator diets can be reconstructed using mixing models that estimate the proportional contribution of each dietary source to the total diet (Phillips et al., 2014). Analysis of δ^13^C ratios has been used to assess the importance of detrital food webs in maintaining populations of generalist predators during time periods when herbivore prey is absent (Albers, Schaefer, & Scheu, 2006). SIA may also enable researchers to track seasonal changes in predator diets, and may help determine the time period when predators prey on a specific prey (McNabb, Halaj, & Wise, 2001). Birkhofer, Fliessbach, Wise, and Scheu (2011) showed that organic management of wheat fields strengthens the trophic link between generalist predators and herbivorous prey compared to conventional management.

Discrimination factors are key assumptions to interpret trophic positions, but may be species‐specific and depend on factors such as tissue type, nutritional status, habitat, and level of omnivory. Most published studies rely on average values for discrimination factors provided in the literature, which may lead to biased conclusions (Caut, Angulo, & Courchamp, 2009). SIA cannot pinpoint the specific identity of prey, as it only distinguishes between dietary sources with contrasting isotope signatures. Still, prey that differentially uses aquatic and terrestrial resources (Paetzold, Lee, & Post, 2008) or C_3_ and C_4_ plants (Albers et al., 2006) can be distinguished, but without the ability to provide quantitative estimates.

### 7.2 Future refinements and improvements

The main challenge of SIA is to identify and control the sources of variation in isotopic signatures to correctly estimate trophic relationships, particularly if samples are collected over a larger geographic range (Birkhofer et al., 2016). Researchers should incorporate laboratory measurements of fractionation factors in their study system if no appropriate estimates exist (Martínez‐del‐Río, Wolf, Carleton, & Gannes, 2009). When possible, tissue types with a known turnover rate should be used (Perkins et al., 2013). Performing SIA for different tissue types may, however, be profitable as this may also provide information on diet changes (Belivanov & Hamback, 2015).

## 8 Fatty Acid Analyses

Two types of fatty acids (FAs) generally prevail in living cells: Phospholipid FAs are the main components of cell membranes, whereas neutral lipid FAs occur in eukaryotes within the fat body. Particular FAs are only synthesized by specific organism groups, mostly bacteria, fungi, algae, and plants, and can, therefore, serve as biomarkers (Traugott et al., 2013). Animal consumers assimilate these FAs from food and incorporate them in their fat body. Such FAs in consumer tissue therefore reflect the consumer's diet (Ruess & Chamberlain, 2010). Similar to SIA methods, FA analysis is particularly suitable for predation studies that address links between predators and groups of prey (Figure 1, e.g., Pollierer, Scheu, & Haubert, 2010). However, the existence of specific biomarker FA's allows for a better resolution of the consumed prey.

### 8.1 Applications and advantages and disadvantages

#### 8.1.1 Analysis of fatty acid composition

FA analyses provide an indirect and time‐integrated picture of feeding strategies and consequently contribute to disentangling food web structure rather than assigning prey types to predator species directly. Haubert, Pollierer, and Scheu (2011) showed that changes in diet of consumers are reflected in their FA patterns after 1 day and are still detectable after 14 days. Among the recent methods for assessment of predation, FA analysis, therefore, allows for a rather middle‐termed tracing of feeding strategies.

FA analysis does not provide quantitative data on feeding rates. It primarily allows for comparative studies analyzing relative feeding strategies of predators in different habitats, under different environmental conditions as well as trophic niche differentiation between coexisting species (Ferlian, Scheu, & Pollierer, 2012). Factors such as environmental conditions (van Dooremalen, Pel, & Ellers, 2009), life stage (Ferlian et al., 2012), and starvation level (Haubert, Haggblom, Scheu, & Ruess, 2004) can bias FA compositions. Most terrestrial studies using FA analysis have so far been applied to rather cryptic systems such as soil habitats (Ferlian et al., 2012; Ruess, Haggblom, Langel, & Scheu, 2004). FA analyses have the advantage of being a rather low‐cost laboratory method compared to for example DNA‐based approaches.

#### 8.1.2 ^13^C fatty acid analysis

Measuring ^13^C/^12^C ratios of individual FAs in predators, in addition to their composition, provides information on the origin of carbon assimilated by the consumer. This may help to overcome the weakness of missing FA biomarkers for certain basal resources (Traugott et al., 2013). So far, most of the studies in terrestrial ecosystems used this approach to investigate trophic interactions in Collembola (Chamberlain, Bull, Black, Ineson, & Evershed, 2004, 2006), but measures of FA ^13^C/^12^C ratios in predators are still rare (but see Haubert et al., 2009). Suitable fields of application include systems with resources of contrasting natural ^13^C signatures due to isotopic fractionation during photosynthesis (C_3_ and C_4_ plants). Furthermore, labeling specific resources, such as leaf litter, with ^13^C allows tracing energy flow over particular marker FAs through the whole food web (Ruess & Chamberlain, 2010). An advantage of the labeling approach is that any isotopic fractionation is negligible (see section 6) as the strong ^13^C labeling signal generally drowns isotopic fractionation.

### 8.2 Future refinements and improvements

To quantify predation, studies on factors, such as lipid metabolism, that influence FA patterns and ^13^C signatures are needed to develop calibration coefficients with which FA data can be corrected (Traugott et al., 2013). The lack of marker FAs, especially for organisms at higher trophic levels, requires exploration of FA patterns of animal consumers in detail to extend the collection of reliable marker FAs (Ruess & Chamberlain, 2010). In the case of ^13^C FA analysis, more studies are needed to distinguish ^13^C signatures of different carbon sources and to relate them to consumer diets.

## 9 Conclusions

The selection of appropriate methods to answer specific questions about predation in the field (e.g., full food web structure vs. suppression of a single pest prey) should be driven by the required level of predator and prey identity (whole communities vs. single or few species, see Figure 1), the needs for qualitative or quantitative predation data (Figure 1) and the appropriate predator traits such as body size and mobility (Table 1). Figure 1 and Table 1 direct the reader toward the most appropriate subset of methods and the respective text section can then be used to identify key references with more detailed advice on the application of methods. We also emphasize how combinations of the introduced methods, due to their individual advantages, can be used to maximize the knowledge gain from predation studies. Studies that utilized cages in combination with diagnostic PCR for example highlighted the importance of major predator groups and certain predator species as antagonists of agricultural pests (Birkhofer, Gavish‐Regev et al., 2008; Furlong, 2015; Roubinet et al., 2015). Apart from only addressing the role of major predator groups by cage treatments, the authors were able to identify the most important trophic links on a species level by molecular gut content analyses. Stable isotope approaches have been combined with diagnostic PCR to identify trophic niche separation between co‐occurring predator species in glacier forelands (Raso et al., 2014). The joint use of the two methods allowed the authors to address both the utilization of a common decomposer prey (by means of PCR) and the importance of intraguild prey (by use of SIA) in arthropod predators.

Predation is without doubt among the most important biotic interactions in terrestrial ecosystems. Selecting the appropriate method according to criteria such as the level of predator and prey organization (e.g., single species or communities), the need for qualitative or quantitative estimates of predation, and according to constraints due to predator traits is a crucial first step to design field studies on predation. Utilizing the array of existing methods, particularly by combining advantages of individual methods will considerably improve our future knowledge of the role of predation events for human societies. Here, we provide a first comprehensive overview of the advantages and disadvantages of available methods for field studies of predation in invertebrate predators of terrestrial ecosystems and give recommendations to assist researchers during the selection of methods.

## Author Contributions

All authors developed the idea and structure of the publication at a joint workshop at SLU Uppsala that was organized by CB and MJ. KB acted as lead author and received text sections from the individual experts for each method.
